# Supplementary material for: Potential Antagonistic Bacteria against Verticillium dahliae Isolated from Artificially Infested Nursery
Source: Cells. 2021 Dec 20;10(12):3588. doi: 10.3390/cells10123588 (PMC8699867; doi:10.3390/cells10123588)
Supplement: Supplementary file 1 [file cells-10-03588-s001.zip › cells-1464179-supplementary.pdf]

Supplementary Materials

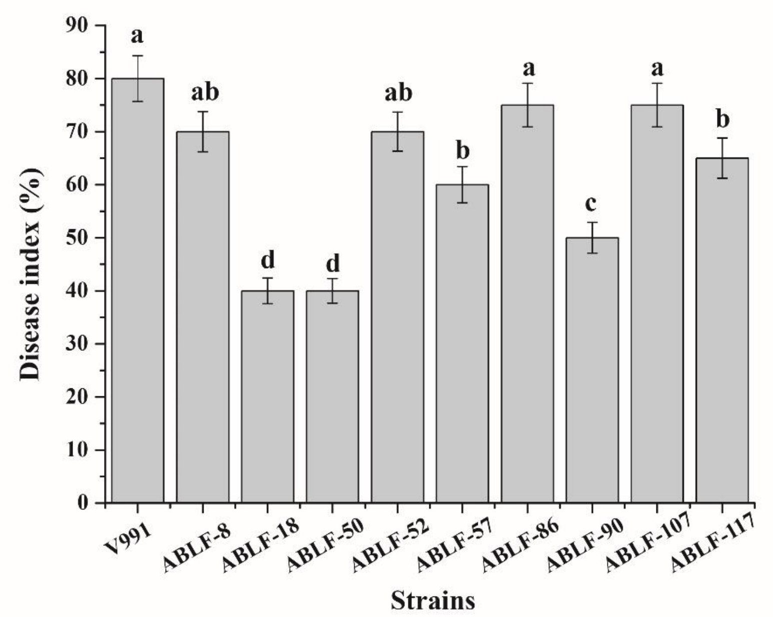

Figure S1. Biocontrol assessment of antagonistic bacterial strains against *V. dahliae* strain V991. Cotton seedlings in pots were inoculated with a candidate bacterial strain. After 24 h, root were dipped in a conidial suspension of *V. dahliae*. At 14 dpi, symptoms were recorded and the disease index was calculated based on the means for three independent experiments. Different letters above bars for a carbon source indicate a significant difference among strains ($P < 0.05$).
